# Metastatic breast cancer cells are selectively dependent on the mitochondrial cristae-shaping protein OPA1

**DOI:** 10.1038/s41419-025-07878-5

**Published:** 2025-07-21

**Authors:** Antigoni Diokmetzidou, Aurora Maracani, Anna Pellattiero, Mauricio Cardenas-Rodriguez, Erwan A. Rivière, Luca Scorrano

**Affiliations:** 1 Department of Biology, University of Padova, Padova, Italy; 2 Veneto Institute of Molecular Medicine, Padova, Italy

**Keywords:** Energy metabolism, Apoptosis, Targeted therapies

## Abstract

In breast cancer, the inner mitochondrial membrane fusion protein Optic Atrophy 1 (OPA1) is upregulated and its inhibition reverses acquired chemoresistance. However, it remains unclear whether OPA1 inhibition also targets normal breast cells. We show that OPA1 upregulation is a hallmark of metastatic breast cancer cells, which are selectively susceptible to OPA1 inhibition compared to isogenic normal or localized tumor cells. In an isogenic model spanning normal, transformed, and metastatic breast cancer cells, levels of Mitofusin 1 (MFN1) progressively declined while dynamin related protein 1 (DRP1) became increasingly active, correlating with fragmented mitochondria during cancer progression. Meanwhile, OPA1 levels were elevated in invasive cells characterized by mitochondrial fragmentation, tight cristae, and high respiration. OPA1 deletion selectively reduced metastatic cells mitochondrial respiration, proliferation, and migration. Specific OPA1 inhibitors MYLS22 and Opitor-0 diminished migration and increased death of metastatic cells, underscoring OPA1 as a selective vulnerability of metastatic breast cancer.

## Introduction

Metastatic disease is a major obstacle in curing breast cancer, hindering the positive outcome of current treatments and increasing the mortality rate in all breast cancer subtypes. Triple-negative breast cancer (TNBC) that lacks estrogen and progesterone receptors as well as human epidermal growth factor receptor 2 (HER2) is the most aggressive subtype, with high metastatic and recurrence rates. Aggressively metastatic TNBC cells are also metabolically plastic, adapting their metabolism to microenvironmental changes and complicating the development of an effective treatment. Often, increased metastatic potential is coupled to increased mitochondrial oxidative phosphorylation (OXPHOS) activity, pointing to a role for mitochondria in the adaptability of TNBC [1–4]. Treatments targeting OXPHOS, such as IACS-010759 have therefore been put forward. However, they lack tumor specificity and display toxicity that precludes advancement past phase I [5].

The OXPHOS system is modulated by changes in mitochondrial shape, regulating fuel utilization and mitochondria-dependent cell growth [6]. Mitochondrial dynamics i.e., the constant cycle between fusion and fission events that dictate mitochondrial morphology, is involved in many facets of tumor initiation and progression, including proliferation, bioenergetics, apoptosis, and cell migration [7, 8]. Moreover, mitochondrial dynamics are necessary for metabolic adaptation [9].

Mitochondrial fission is regulated by the dynamin-related protein 1 (DRP1) that binds to its adapters on the outer mitochondrial membrane (OMM), mitochondrial fission factor (MFF), mitochondrial division 49 and 51(MiD49 and 51), and fission 1 (Fis1). The role of fission and its machinery in the progression of breast cancer is rather ambiguous. DRP1 expression is reported to be upregulated in human invasive breast carcinomas [10] and metastatic lesions in the brain and lymph nodes [10, 11] compared to normal breast tissues or non-invasive breast cancer cells. On the other hand, gene expression data from patients revealed that increased expression of genes associated with mitochondrial fission correlated with improved survival in human breast cancer [12]. Both impairing or enforcing mitochondrial fission genetically or pharmacologically was shown to suppress tumor growth and metastasis [11–13]. Similarly, mitochondrial fission was shown to either promote or inhibit apoptosis in numerous TNBC cells [12, 14].

Mitochondrial fusion is controlled by the GTPases Mitofusin (MFN) 1 and 2 in the OMM and optic atrophy 1 (OPA1) in the inner mitochondrial membrane (IMM). Independent of its role in fusion, OPA1 also controls apoptotic cristae remodeling by keeping the cristae junctions (CJ) tight through the formation of high molecular weight oligomers [15]. Moreover, by regulating IMM morphology, OPA1 favors assembly of the respiratory chain supercomplexes and respiratory efficiency [6]. OPA1 levels correlate with worse breast cancer prognosis [16, 17] and are increased in residual TNBC following treatment with DNA-damaging chemotherapy [18]. OPA1 deletion reduces tumor angiogenesis, growth, and metastasis in breast cancer [17, 19], and its pharmacological inhibition restores the sensitivity to chemotherapy [18]. Thus, OPA1 appears as a potential therapeutic target for TNBC. However, it is unclear whether OPA1 is specifically upregulated in metastatic breast cancer compared to normal breast cells, questioning whether OPA1 displays a large enough therapeutic window to be safely targeted in TNBC.

With this question in mind, we used an isogenic model of human breast cancer progression to systematically profile mitochondrial dynamics. We report that OPA1 is selectively upregulated in metastatic breast cancer irrespective of the observed fragmented mitochondrial phenotype, and that its genetic or pharmacological inhibition selectively limits proliferation and migration in metastatic over normal and transformed breast cells.

## Materials and methods

### Cell culture

MCF10A, MCF10AT1, MCF10CA1h, and MCF10CA1a cells were purchased from the Animal Model and Therapeutic Evaluation Core (AMTEC), Karmanos Cancer Institute, Wayne State University, USA. All cells were cultured at 37 °C in a 5% CO_2_ atmosphere in Plasmax [20] supplemented with 1% penicillin/streptomycin (Gibco, Cat. #15140), 10 µg/ml insulin (Sigma-Aldrich, Cat. #I6634), 20 ng/ml Recombinant Human EGF (PeproTech Ich., USA, Cat. #AF-10015) and 0.5 µg/ml Hydrocortisone (Sigma-Aldrich, Cat. #H0888) in the case of MCF10A cells. Cells were cultured for at least 10 passages in Plasmax before any experiment. Where indicated, cells were switched to DMEM supplemented with 10% fetal bovine serum (FBS, Gibco, Cat. #A5256701), 1% non-essential amino acids (MEM NEAA, Gibco, Cat. #11140035), and 1% penicillin/streptomycin/glutamine (Gibco, Cat. #15140-122 and Cat. #25030-024) and cultured for 10 passages before experiments were performed.

Lenti-X 293T cells were cultured at 37 °C in a 5% CO_2_ atmosphere using Dulbecco’s Modified Eagle Medium (DMEM, Gibco, Cat. #12800082) supplemented with 10% fetal bovine serum (FBS, Gibco, Cat. #A5256701), 1% non-essential amino acids (MEM NEAA, Gibco, Cat. #11140035), and 1% penicillin/streptomycin/glutamine (Gibco, Cat. #15140-122 and Cat. #25030-024). All cells were regularly tested for Mycoplasma contamination.

### Generation of *OPA1*^−/−^ cell lines

To generate stable *OPA1*^−/−^ 10 A, AT1, CA1h and CA1a cell lines, we targeted by CRISPR-Cas9 genomic *OPA1*. Guide RNAs targeting exon 26 (clone 1) or exon 2 (clone 2) of OPA1 were designed using the CRISPR online design tool CHOPCHOP V2.0 (https://chopchop.cbu.uib.no/). Designed guide RNAs had no off-target sites and were ordered from Eurofins Genomics. Oligomers were phosphorylated and annealed into duplexes using T4 Polynucleotide kinase (New England Biolabs (NEB), Cat. #M0201L). Duplexes were then inserted into BsmBI-v2 (NEB, Cat. #R0739L) digested lentiCRISPR v2 plasmid (Addgene, Plasmid # 52961) using a T7 DNA Ligase (New England Biolabs (NEB), Cat. #M0318S). The DNA product was transformed into chemically competent XL10-Gold bacteria and grown on Ampicillin resistance plates for selection. Successful cloning of guide RNAs into the lentiCRISPR v2 plasmid was confirmed by genomic sequencing (Eurofins, Mix2Seq kit). The generated Cas9-gRNAs plasmid or the lentiCRISPR-EGFP_sgRNA1 (Addgene Plasmid #51760), psPAX2 (Addgene, Plasmid #12260), and pMD2.G (Addgene, Plasmid # 12259) were co-transfected into Lenti-X 293T cells using Linear Polyethylenimine Hydrochloride (PEI max, Polysciences, Cat. #24765) to produce Lentiviruses. Supernatant containing viruses were used to infect the cells, which were then selected using puromycin (5 μg/ml) for 96 h.

### OPA1 inhibitors

The specific OPA1 GTPase inhibitors MYLS22 [19] and Opitor-0 were synthesized, purified, and chemically characterized in-house as described in ref. [21] and used at the concentrations detailed in the figure legends.

### Mitochondrial morphology analysis

Mitochondrial morphology was analyzed in live cells cultured in Plasmax using an Operetta CLS high content analysis system imaging (Revvity) in confocal mode equipped with a C-Apochromat 63X/1.15 W UV VIS IR water immersion objective, temperature, and CO_2_ control. Cells were transduced with Lenti-virus carrying mitochondrially targeted YFP [22] and after 48 h selected by puromycin addition in the media. OPA1^−/–^ cells were incubated with 50 nM Mitotracker Deep Red (Invitrogen, Cat. #M22426) in serum-free Plasmax for 30 min and then the media were replaced with Plasmax. Multiple wells were analyzed per condition with at least 40 fields per well in 3 or more biological replicates. Images were analyzed using Harmony 5.1 or Columbus softwares (Revvity). Mitochondrial length, Circularity (4π*area/perimeter^2^), Aspect ratio (Major/Minor axis), Aspect ratio/Circularity, and Form Factor (perimeter^2^/4π*area) were used as readout parameters.

### Western blot analysis

Cells were lysed in RIPA buffer (1% NP-40, NaCl 150 mM, DOC 12.75 mM, SDS 3.5 mM, Tris 50 mM) and protein content was quantified using Pierce BCA Protein Assay Kit (Thermo Fisher Scientific, Cat. #23225). Samples were prepared using NuPAGE LDS Sample Buffer (Thermo Scientific, Cat. #NP0008) with 2.5% β-mercaptoethanol (BME) (Sigma, Cat. #M3148) and heated at 95 °C for 5 min. Equal amounts of protein were electrophoresed in ExpressPlus PAGE 4–12% gel (GenScript, Cat. #M41212S) and transferred to nitrocellulose membrane (Amersham, Cat. No.10600001) by a constant voltage of 30 mV for 2 h. Membranes were blocked with 5% non-fat dry milk or 5% bovine serum albumin (Capriconrn Scientific, Cat. #BSA-1T) in TRIS-Buffered Saline with 0.5% Tween 20 and incubated overnight with the indicated primary antibodies at the indicated dilution (Supplementary Table 1) at 4 °C. After washing, the appropriate HRP-conjugated secondary antibody (Li-Cor, Cat. #926-800010 or 926-80011, Supplementary Table 1) was added at 1:10,000 dilution for 1 h at room temperature. Chemiluminescence was detected using Immobilon Crescento Western HRP Substrate I Millipore, Cat. #WBLUR0500). Images were acquired using an iBright™ FL1500 Imaging System (Thermo Fisher Scientific). Densitometric analysis of the immunoblots was performed using FIJI (NIH) [23].

### Chemical crosslinking

In vivo chemical crosslinking with disuccinimidyl suberate (DSS) (Thermo Fisher Scientific, Cat. #A39267) was performed according to the manufacturer’s protocol with some modifications. Cells were washed three times in phosphate-buffered saline (PBS), and incubated with freshly prepared 1 mM DSS or DMSO in PBS for 1 h at 4 °C. The reaction was quenched by 100 mM Tris (pH 7.5) for 30 min at room temperature. The samples were dissolved in NuPAGE™ LDS sample buffer (Thermo Scientific Cat. #NP0008) and analyzed by western blotting using Nupage 3–8% Tris Acetate gels (Thermo Scientific, Cat. #EA03752BOX).

### mtDNA quantification

Total DNA was extracted using Puregene Core Kit A (Qiagen, Cat. #1042601) according to the manufacturer’s instructions. Quantitative real-time PCR was performed in a Qiaquant 384 5plex thermocycler (Qiagen) using 4 ng of DNA with SYBR green (Applied Biosystem, Cat. #4367659) and specific primers (250 nM, (Supplementary Table 2). Relative copy number was calculated using the relative 2^−∆∆Ct^ method.

### Transmission electron microscopy

Cells were fixed with 2.5% glutaraldehyde in 0.1 M sodium cacodylate pH 7.4 overnight at 4 °C. Samples were postfixed with 1% osmium tetroxide plus potassium ferrocyanide 1% in 0.1 M sodium cacodylate buffer for 1 h at 4 °C. After three water washes, samples were dehydrated in a graded ethanol series and embedded in an epoxy resin (Sigma–Aldrich). Ultrathin sections (60–70 nm) were obtained with Leica Ultracut EM UC7 ultramicrotome, counterstained with uranyl acetate and lead citrate and viewed using a Tecnai G2 (FEI) transmission electron microscope operating at 100 kV equipped with a Veleta (Olympus Soft Imaging System) digital camera at the BioImaging Facility of the Dept. of Biology of the University of Padua. At least 3 biological replicates were processed and ~150 images were taken per sample. Images were quantified using Fiji in a blinded manner.

### Seahorse respirometry

Cells were seeded in Plasmax in an XF24 cell culture microplate in a density of 40,000–50,000 cells/well. The next day the medium was changed to Plasmax without phenol red, sodium bicarbonate and FBS and plates were incubated at 37 °C in a CO_2_-free incubator for 1 h. Oxygen consumption rate (OCR) was measured using an Agilent Seahorse XFe24 Analyzer at basal conditions and after the addition of Oligomycin (1.5 µM; Sigma Aldrich, Cat. #O4876), FCCP (1 µM; Sigma Aldrich, Cat. #C2920), and Rotenone (1 µM; Sigma Aldrich, Cat. #R8875) and Antimycin A (1 µM; Sigma Aldrich, Cat. #A8674). Measurements were taken over 3 min intervals, proceeded by 30 s mixing and 2 min incubation. Following the completion of the assay, Hoechst (Thermo Scientific, Cat. #H3570) was added to the cells and the whole plate was imaged by Operetta CLS using a 10× objective at 37 °C and 5% CO_2._ The cell number per well was calculated using Harmony (Revvity). All data were normalized and analyzed using Seahorse Analytics software (Agilent Technologies).

### Migration assay

To estimate the migratory capacity of the cells the classical scratch wound assay was performed in a high-throughput mode. Cells were plated in 48-well for 48 h and then the head of a 200 μL tip was used to perform a wound. The cells were followed for 20 h and imaged every 4 h and 30 min using Operetta CLS (Revvity) equipped with a 10× air objective, at 37 °C and 5% CO_2_. The cell-free area (gap) was detected using Harmony software and the coverage was calculated by dividing the gap at a certain time point to the initial gap. In the case of MYLS22 and Opitor-0, the treatment was added directly after the scratch.

### Cell proliferation

Cell proliferation was determined by quantification of Phospho-Histone H3 (P-HH3) positive normalized on the total nuclei number. Cells plated in 96-well plates (Greiner Bio-One, Cat. #675090) were fixed with 4% paraformaldehyde for 15 min at room temperature, permeabilized with 1% Triton solution in PBS, and blocked with 5% BSA solution in PBS for 1 h at RT. Primary anti-P-HH3 (Cell Signaling Technology, Cat. #53348) and Ki67 (Thermo Scientific, Cat. #MA5-14520) antibodies were added at 1:200 and incubated overnight in 5%BSA in PBS. The corresponding isotype matched secondary fluorescent antibodies Alexa Fluor 594 (Thermo Fisher Scientific), were incubated for 1 h at room temperature. Nuclei were stained with Hoechst (Thermo Scientific, Cat. #H3570). The samples were imaged using an Operetta CLS (Revvity) system equipped with a 10× air objective and analyzed using Harmony 5.1 (Revvity).

### Cell death analysis

Cell death was assessed using the Annexin-V Apoptosis detection kit (Invitrogen, Cat. #BMS500FI) following the manufacturer’s instructions. Annexin V-FITC and PI staining were detected by flow cytometry using a LSRFortessa X-20 (BD Biosciences). Cell viability was measured as the percentage of Annexin-V and PI-negative events. Alternatively, cell death was quantified by quantifying the number of Sytox Green (ThermoFisher, Cat. #7020) positive cells normalized on the total amount of cells using an Operetta CLS system equipped with a 10× air objective at 37 °C and 5% CO_2_ and Harmony software for analysis.

### Statistics

All data are presented as mean ± standard error (SEM) of at least three independent experiments. The *N* of independent biological experiments is indicated in the figure legends. Statistical analysis was performed using OriginPro 2024 (Microcal). When analyses were performed between two independent groups, a Mann–Whitney non-parametric test was used. For three or more groups, one-way ANOVA was used to detect the difference among groups with Tukey post hoc test for means comparison for normally distributed data, or Kruskal–Wallis ANOVA with Dunn post hoc test for means comparison if data were not normally distributed. The variance was similar for all the groups tested. The difference between two or more groups was considered significant if *p* ≤ 0.05 (*), *p* ≤ 0.01 (**) or *p* ≤ 0.001 (***).

## Results

### Mitochondrial fragmentation is a hallmark of metastatic breast cancer cells

**Fig. 1:**
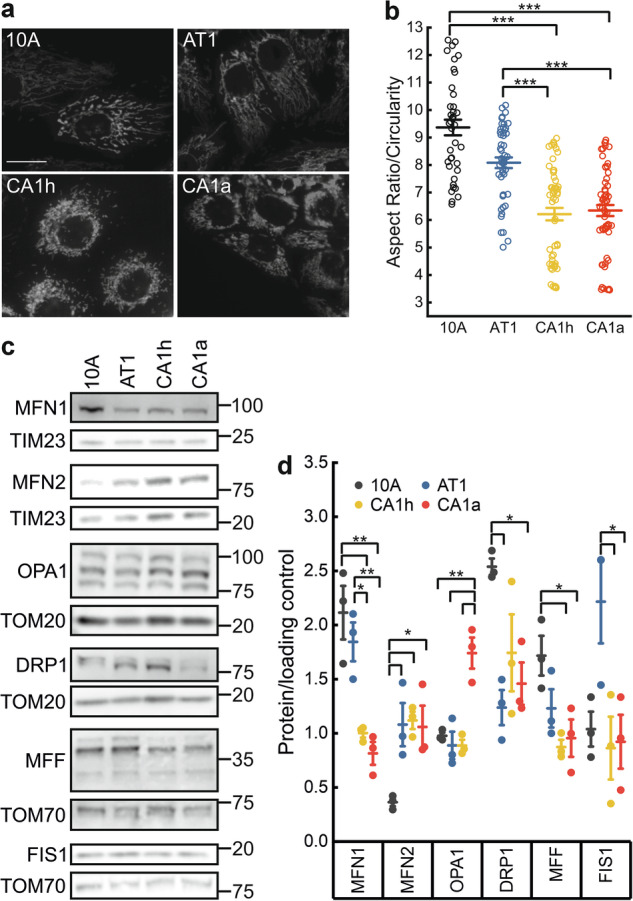
Mitochondria are fragmented in metastatic breast cancer cells. **a** Representative confocal images of lentivirally expressed mtYFP in the indicated isogenic cells. Scale bar: 25 µm. **b** Mean ± SEM of mitochondrial aspect ratio (length/width) normalized by circularity in cells as in a. Each dot refers to the mean of ≥40 imaging fields per well from *n* = 7 independent experiments. ***p < 0.0001 by Kruskal–Wallis ANOVA and Dunn’s post-hoc test. **c** Cells were lysed and equal amounts of proteins (30 µg) were separated by SDS-PAGE and immunoblotted using the indicated antibodies. TIM20, TIM23, and TOM70 were used as loading control. **d** Quantification of the indicated proteins of the dynamics machinery by densitometric analysis as in c. Data are mean ± SEM from *n* = 3 independent experiments. *p ≤ 0.05, **p < 0.01 by One-way ANOVA with Tukey post-hoc test.

To explore the interplay between mitochondrial morphology and metastatic potential, we unbiasedly analyzed by high content imaging the mitochondrial network in an isogenic model of triple negative human breast cancer progression cultured in a human plasma-like medium [20]. This isogenic series derives from the benign immortalized MCF10A cell line of human breast epithelial cells origin. Upon MCF10A transformation with c-Ha-Ras oncogene, the resulting MCF10AT1 (from here on AT1) cells can generate atypical hyperplasia and carcinoma in situ [24]. MCF10CA1a and MCF10CA1h (from here on CA1a and CA1h) originate from the MCF10AT progression but differ in tumorigenicity and invasiveness, CA1a being highly tumorigenic and able to metastasize to distant organs such as lungs and liver, whereas the less metastatic CA1h mostly causes localized tumors [25]. High throughput confocal imaging of mitochondrially-targeted yellow fluorescent protein (YFP) expressed in all the cell lines revealed that the mitochondria of the metastatic cells (CA1h and 1a) were shorter and formed fragmented and less complex networks compared to the elongated tubular mitochondrial networks found in the benign (10A) and non-metastatic (AT1) cells (Fig. 1a, b and Supplementary Fig. 1a, b). By measuring levels of the core mitochondria-shaping proteins in these isogenic cell lines, we found a significant decrease in MFN1 in all transformed cell lines, whereas MFN2 was increased. Moreover, while total levels of DRP1 were unchanged, levels of Ser616 phosphorylation that promote DRP1 activation and mitochondrial fission [26], were elevated in metastatic cells (Supplementary Fig. 1c, d). Indeed, the assembly of DRP1 in fission competent, DSS stabilized tetramers and higher oligomeric states was increased in metastatic breast cancer cells (Supplementary Fig. 1e, f), further explaining the mitochondrial fragmentation observed in these cells. Conversely, OPA1 levels were increased in the highly invasive cell line CA1a where mitochondria appeared fragmented. These data indicate a linear relationship between mitochondrial fragmentation and breast cancer progression but also point out to a selective increase in OPA1 in highly metastatic breast cancer cells.

### Tighter cristae and elevated OPA1 oligomers in metastatic breast cancer cells

**Fig. 2:**
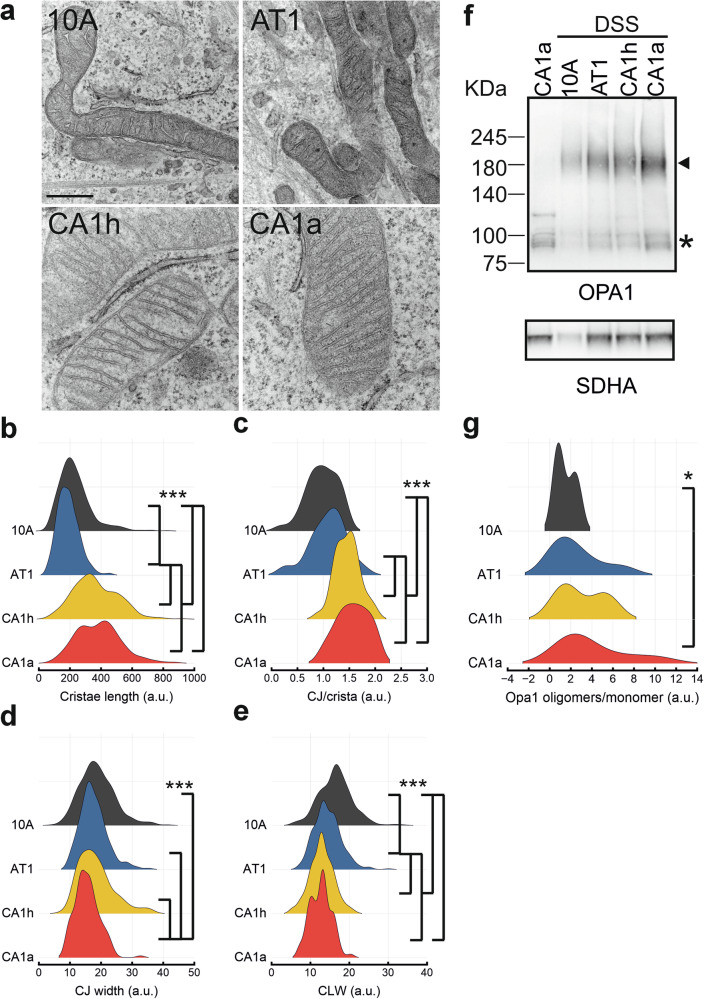
Metastatic cells have tighter cristae and elevated OPA1 oligomers. **a** Representative electron micrographs of mitochondria from 10 A, AT1, CA1h and CA1a cells. Scale bar: 100 nm. Morphometric analysis of cristae length (**b**), cristae junctions (CJ) per cristae (**c**), cristae junctions width (**d**) and cristae lumen width (CLW, e) in mitochondria as in (**a**). Data are mean ± SEM from *n* = 3 independent experiments where >300 cristae from *n* > 32 mitochondria per cell line were analyzed. ***p ≤ 0.001 by Kruskal–Wallis ANOVA and Dunn’s post-hoc test. **f** Cells treated, where indicated, for 1 h with 1 mM DSS were lysed and equal amounts of proteins (30 µg) were separated by SDS-PAGE and immunoblotted using the indicated antibodies. Asterisk: monomeric OPA1; arrow: OPA1 oligomers. **g** Quantification of OPA1 oligomers/monomer ratio by densitometric analysis in *n* = 7 independent experiments as in (**f**). *p ≤ 0.05 by One-way ANOVA with Fisher’s post-hoc test.

We were surprised to observe that levels of the pro-fusion protein OPA1 were increased in the breast cancer cells displaying the most fragmented mitochondrial phenotype. Because OPA1 controls the organization and remodeling of mitochondrial cristae independently from its role in mitochondrial fusion [15], we turned to transmission electron microscopy (TEM) to evaluate whether the changes in mitochondrial ultrastructural organization accompanied the observed fragmentation. By inspecting TME images, we confirmed the fragmentation observed in the metastatic cells compared to their isogenic non-metastatic counterparts (Fig. 2a). Ultrastructurally, cristae appeared tighter, longer and the number of CJ increased in both the metastatic cell lines compared to the isogenic non metastatic cells. Finally, CJ width was significantly reduced in the highly invasive CA1a cells, which displayed the highest levels of OPA1 (Fig. 2b–e). Indeed, levels of OPA1 oligomers that stabilize CJ width were increased in all the transformed cell lines, and most prominently in CA1a cells (Fig. 2f, g). Thus, in metastatic breast cancer cells, active DRP1 in combination with low MFN1 levels tips the balance towards fission, whereas increased OPA1 levels tighten cristae and CJ.

### Transformed TNBC cells rely on OPA1 for efficient mitochondrial respiration

**Fig. 3:**
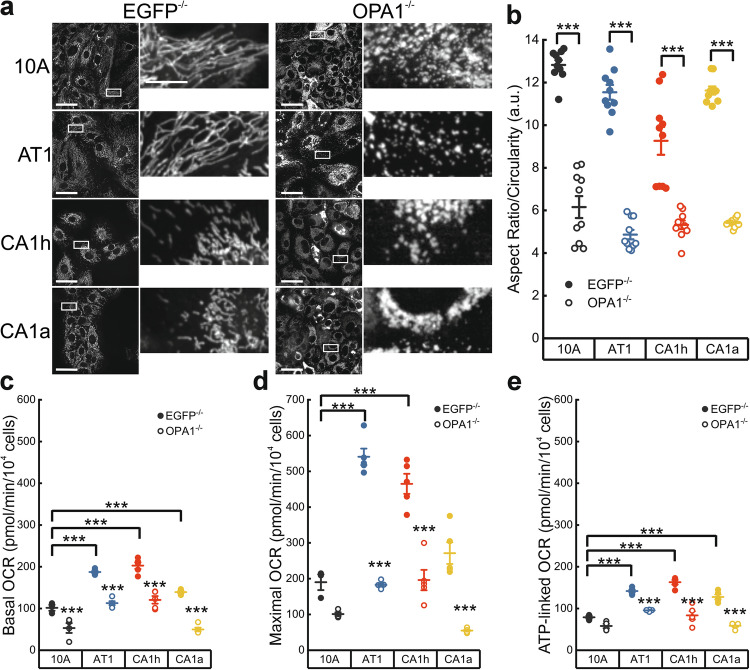
Deletion of OPA1 reduces mitochondrial respiration in transformed but not normal breast cells. **a** Representative confocal images of Mitotracker Deep Red in cells of the indicated genotype. The boxed areas are magnified ×9 in the images on the right. Scale bars: 50 µm (left) and 10 µm (right images). **b** Mean ± SEM of mitochondrial aspect ratio (length/width) normalized by circularity in cells as in (**a**). Each dot refers to the mean of ≥40 imaging fields per well from *n* = 6 independent. ***p < 0.0001 by One-way ANOVA and Tukey’s post-hoc test. **c**–**e** Oxygen consumption rate (OCR) as measured by Seahorse in intact cells of the indicated genotypes. Basal (**c**), maximal (after the addition of 1 µM FCCP) (**d**) and oligomycin (1.5 µM)-sensitive OCR (**e**) are the mean ± SEM from *n* = 3 independent experiments. ***p < 0.001 by One-Way ANOVA with Tukey’s post-hoc test. Where not otherwise indicated the statistical significance of EGFP^−/−^ vs OPA1^−/−^ was calculated.

To delineate the role of OPA1 on the metastatic potential of CA1a cells, we employed CRISPR-Cas9 to knockout (KO) OPA1 in an isogenic breast cell series. Following antibiotic selection and limiting dilution to isolate single-cell clones, we obtained two independent KO clones per isogenic cell line. Immunoblot analysis confirmed the complete loss of OPA1 expression in both clones from each line (Supplementary Fig. 3a). In control clones, generated by puromycin selection of cells transduced with a CRISPR/Cas9-EGFP targeting vector, high-content live cell imaging of lentivirally expressed mtYFP revealed a slight increase in mitochondrial length relative to the parental, unperturbed lines -a change likely attributable to a stress response [27]. Consistent with the known function of OPA1, its deletion (OPA1^–/−^) induced the expected mitochondrial fragmentation in all clones (Fig. 3a, b and Supplementary Fig. 3b). Next, we assessed whether OPA1 deletion impacted the mitochondrial bioenergetics of the isogenic breast cells cultured in Plasmax. Notably, mtDNA content remained comparable in 10 A, AT1, and CA1h cells, whereas it was reduced in the highly metastatic CA1a cells (Supplementary Fig. 3c). Given that the panel of mitochondrial proteins did not indicate a loss of mitochondrial mass in CA1a cells, we believe that the decreased mtDNA levels may result from impaired mitochondrial fusion due to reduced MFN1 levels (Fig. 1c, d). Indeed, mitochondrial fusion is critical for the maintenance of mtDNA [28]. Despite lower mtDNA levels, CA1a cells exhibited significantly higher basal and ATP-linked OCR compared to the non-transformed 10 A cells, while maximal OCR, although increased, did not reach statistical significance (Fig. 3c–f). This observation aligns with the linear correlation between OCR and the stage of breast cancer progression, where transformed cells consistently display higher OCR than non-transformed 10 A cells (Fig. 3c–f). Notably, while OPA1 deletion reduced basal OCR across all cell lines, maximal and ATP-linked OCR were specifically impaired in transformed cells. This suggests that OPA1 is critical for the efficiency of the respiratory chain and mitochondrial ATP production in transformed cells (Fig. 3c–f). In summary, although OPA1 deletion induces extensive mitochondrial fragmentation in all cell lines, it is indispensable for efficient respiration only in transformed breast cancer cells, in contrast to their non-tumor isogenic counterparts.

### Metastatic TNBC migration and proliferation depends on OPA1

**Fig. 4:**
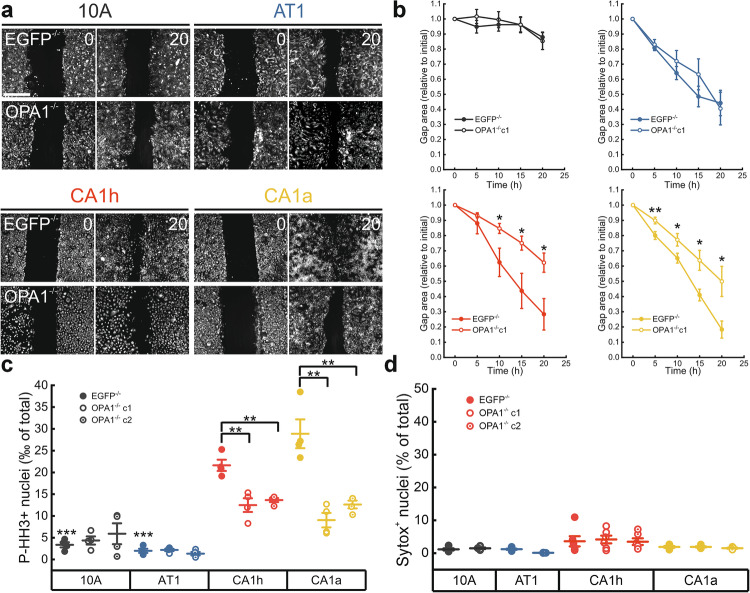
OPA1 deletion limits migration and proliferation selectively in metastatic breast cancer cells. **a** Representative images of Mitotracker Deep Red in cells of the indicated genotype acquired at the beginning (0 h) and after 20 h in a scratch wound assay. Scale bar: 500 µm. **b** Quantification of cellular migration (remaining gap area normalized to initial gap) as in (**a**), at the indicated timepoints. Data are the mean ± SEM of *n* = 5 independent experiments. *p ≤ 0.05, **p < 0.01 by one-way ANOVA with Tukey’s post-hoc test. **c** Quantification of the Phospho-Histone H3 (P-HH3) positive nuclei per total nuclei number in cells of the indicated genotype. Data are the mean ± SEM of *n* = 4 independent experiments. **p < 0.01 EGFP^−/−^ vs OPA1^−/–^ and ***p < 0.001 10 A and AT1 vs CA1a and CA1h by One-Way ANOVA with Tukey’s post-hoc test. **d** Quantification of the Sytox Green positive cells per total cellular number of the indicated genotype. Data are the mean ± SEM of *n* = 6 independent experiments. No statistically significant difference by one-way ANOVA with Tukey’s post-hoc test.

We then investigated whether OPA1 deletion affected key features of these metastatic TNBC cells that contribute to their aggressive phenotype. We therefore measured migration, proliferation, and apoptosis. To evaluate the impact of *OPA1* deletion on the migratory capacity of metastatic cells, we performed a classic scratch assay. As anticipated, benign and non-metastatic cells failed to effectively close the wound, whereas their malignant counterparts covered ~60% more area within the same period (Fig. 4a, b). Notably, deletion of OPA1 significantly reduced the migratory capacity of metastatic cells, while it had no measurable effect on the migration of benign or non-metastatic cells. To determine if the observed reduction in migration was attributable to slower proliferation or increased cell death, we examined both parameters. Phospho-Histone H3 (P-HH3) staining indicated that *OPA1* deletion reduced proliferation of both metastatic cell lines, whereas it did not affect it in benign and non-metastatic cells (Fig. 4c). Conversely, we did not observe any effect on cell death upon OPA1 deletion in any of the analyzed cell lines, suggesting that the observed changes in metastatic TNBC cells migration and proliferation are not caused by loss of viability upon OPA1 deletion (Fig. 4d). Altogether, these findings highlight that TNBC cells depend on OPA1 for sustaining their proliferative and migratory phenotype.

### OPA1 inhibitors selectively target metastatic TNBC cells

**Fig. 5:**
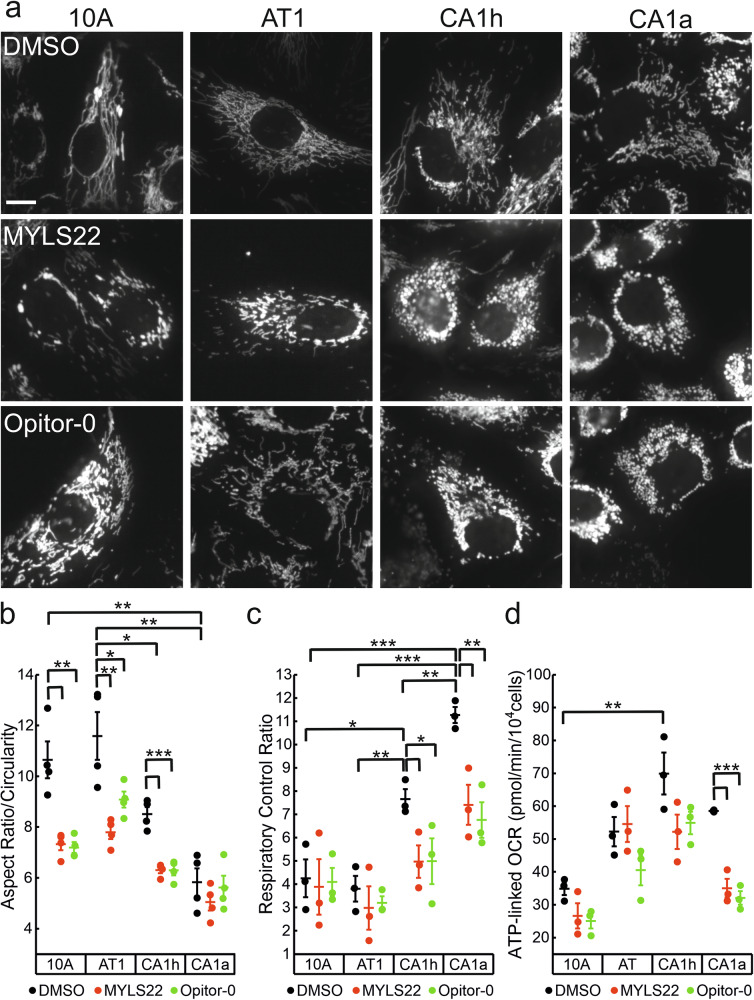
OPA1 inhibition reduces mitochondrial respiration selectively in metastatic breast cancer cells. **a** Representative confocal images of lentivirally expressed mtYFP in the indicated isogenic cells treated with MYLS22 (50 μM), Opitor-0 (50 μM), or DMSO for 24 h. Scale bar: 10 µm. **b** Mean ± SEM of mitochondrial aspect ratio (length/width) normalized by circularity from *n* = 4 independent experiments as in (**a**), where ≥320 fields were imaged. *p ≤ 0.05, **p < 0.01 ***p < 0.001 by one-way ANOVA with Tukey’s post-hoc test. Quantification of respiratory control ratio (RCR) calculated as State 3/State 4 respiration (**c**) and ATP-linked OCR (**d**) measured by Seahorse of the indicated cell lines after incubation with MYLS22 (50 μM), Opitor-0 (50 μM) or DMSO for 6 h. *p < 0.01, **p < 0.01, ***p < 0.001 by one-way ANOVA with Tukey’s post-hoc test. Data are the mean ± SEM of *n* = 3 independent experiments.

**Fig. 6:**
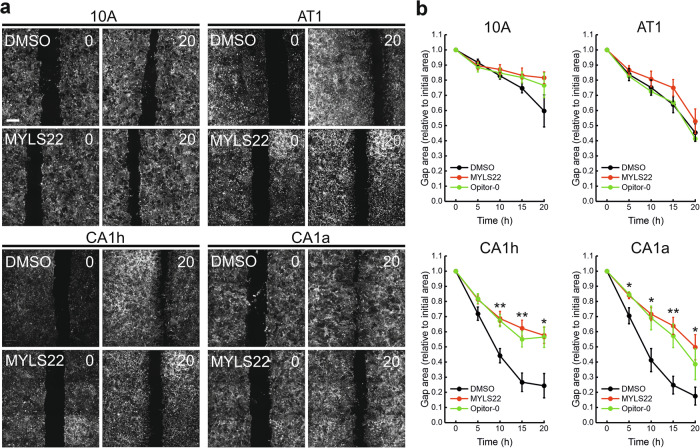
OPA1 inhibition reduces migration selectively in metastatic breast cancer cells. **a** Representative image of Mitotracker Deep Red in the indicated cells at the beginning (0 h) and after 20 h in a scratch-wound assay. The cells were treated with MYLS22 (50 μM), Opitor-0 (50 μM), or DMSO at the beginning of the assay. Scale bar: 500 μm. **b** Quantification of cellular migration (remaining gap area normalized to initial gap) as in (**a**), at the indicated timepoints. Data are the mean ± SEM of *n* = 6 independent experiments. *p ≤ 0.05, **p < 0.01 DMSO vs inhibitors by one-way ANOVA with Tukey’s post-hoc test.

**Fig. 7:**
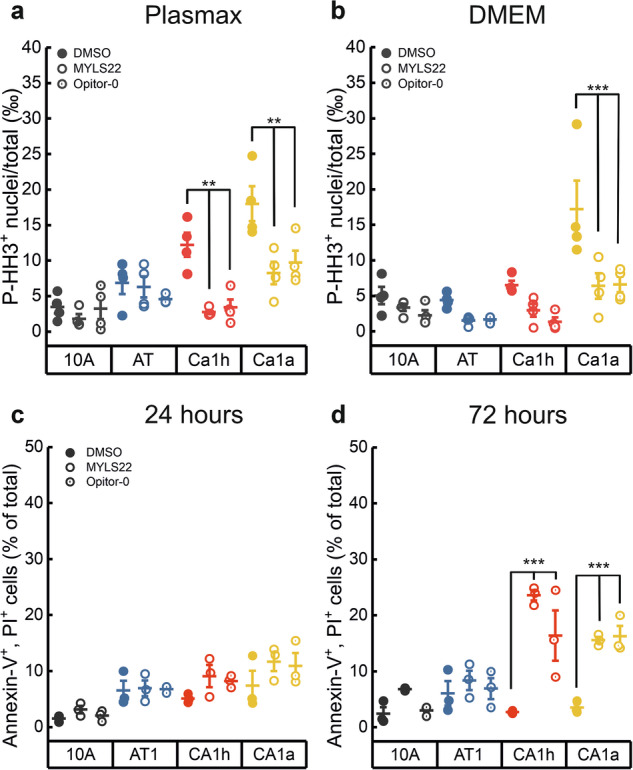
OPA1 inhibition reduces proliferation selectively in metastatic breast cancer cells. Quantification of the P-HH3 positive nuclei per total number of the indicated cells cultured in Plasmax (**a**) or DMEM (**b**) medium and incubated with MYLS22 (50 μM), Opitor-0 (50 μM) or DMSO for 24 h. Data are the mean ± SEM of *n* = 4 independent experiments. **p ≤ 0.01, ***p < 0.001 by one-way ANOVA with Tukey’s post-hoc test. Quantification of Annexin V and Propidium Iodide (PI) positive cells per total number of the indicated cells incubated with MYLS22 (50 μM), Opitor-0 (50 μM) or DMSO for 24 h (**c**) or 72 h (**d**). Data are the mean ± SEM of *n* = 3 independent experiments. ***p < 0.001 by one-way ANOVA with Tukey’s post-hoc test.

Comforted by the results indicating that metastatic cells depend on OPA1, we used small-molecule inhibitors of OPA1 developed in our lab to verify whether OPA1 holds potential as a selective target in metastatic cells. We capitalized on the prototypical first-generation OPA1 inhibitor MYLS22 that is effective against TNBC in vitro and in vivo [17–19] and on Opitor-0, a more efficient inhibitor generated from MYLS22 by structure-activity relationship analysis [21]. We first evaluated the effects of these inhibitors on mitochondrial morphology and function. After 24 h of treatment, mitochondrial length in benign, non-metastatic, and mildly metastatic CA1h cells appeared significantly shorter, while no obvious differences were noted in the highly metastatic CA1a cells, likely due to their preexisting punctate morphology (Fig. 5a, b). Functionally, already after 6 h of treatment MYLS22 or Opitor-0 reduced respiratory efficiency in metastatic cells, without significantly affecting OXPHOS function in non-metastatic cells (Fig. 5c, d). These results recapitulate the results obtained with genetic OPA1 deletion and highlight the dependency of metastatic cells on OPA1 for their mitochondrial function. Tracking migration of the panel of cells treated for 24 h with MYLS22 or Opitor-0 revealed a marked decrease in the wound closure by the metastatic CA1h and CA1a cells, whereas we did not observe any effect on the normal 10A or the non-metastatic transformed AT1 cells (Fig. 6a, b). Similar to what was observed upon OPA1 deletion, these inhibitors affected proliferation only of the highly metastatic CA1a cells (Fig. 7a). Because the effect on proliferation of cells cultured in Plasmax could be due to impaired utilization of carbon sources in this non-conventional medium, we also measured proliferation in standard high glucose DMEM. Upon treatment with MYLS22 and Opitor-0, staining of CA1a cells with the proliferation marker P-HH3 was similarly reduced (Fig. 7b). Furthermore, we excluded that the reduction of proliferation observed after 24 h of treatment could be a side effect of increased apoptosis. Indeed, we did not observe cell death at 24 h in any of the cell lines tested. After 72 h of treatment, we observed a small but significant increase in apoptosis of the metastatic CA1h and CA1a cells (Fig. 7c, d). Collectively, these findings indicate that pharmacological inhibition of OPA1 with MYLS22 or Opitor-0 selectively impairs migration and proliferation of metastatic cells without impacting benign or non-metastatic cells or causing cell death.

## Discussion

Despite recent therapeutic advances, metastatic TNBC continues to have a poorer prognosis than other subtypes because of its aggressive, relapsing behavior and the lack of targeted treatments [29]. Mitochondrial dynamics and OPA1 in particular have emerged as promising targets in TNBC. Notably, OPA1-targeting drugs developed in our laboratory have demonstrated efficacy in both orthotopic and patient-derived xenograft models of TNBC [17, 18]. However, it remained unclear whether these agents selectively target metastatic cells to mitigate their aggressive phenotype. Using an isogenic cell model series that spans normal breast epithelial cells to aggressively metastatic TNBC, we show that metastatic cells are selectively addicted to OPA1. Both genetic ablation and pharmacological inhibition of OPA1 significantly impair the proliferation and migration of metastatic TNBC cells, without inducing cell death or affecting normal or transformed isogenic cells.

Addressing whether a targeted therapy can selectively act on a subtype or a specific stage of cancer is complicated by the vast heterogeneity of available models. Most cancer cell lines are derived from a single patient and are therefore largely divergent genetically, epigenetically, and metabolically from the tissue of origin. Patient-derived xenografts, while approximating clinical reality and serving as invaluable tools for personalized medicine, are even more heterogeneous. To dissect the role of mitochondrial dynamics at different stages of TNBC, we elected to use a well-characterized isogenic series comprising normal, transformed, and locally as well as highly metastatic breast cells [24, 25]. The advantage of using isogenic cell lines is that a similar genetic and epigenetic landscape allows us to isolate and assign the specificity of the observed effects to the targeted gene. Furthermore, we switched from classic high-glucose, unphysiological tissue culture media to quasi-physiological Plasmax, whose solutes, cofactors, metabolites, and carbon sources closely mirror those in human plasma. We believe that culturing cancer cells in a less artificial environment is paramount when addressing the role of mitochondrial dynamics and bioenergetics, which are largely dependent on fuel availability and type [30].

Our profiling of morphology and shaping proteins revealed that the mitochondrial network becomes increasingly fragmented as cells progress toward aggressively metastatic TNBC. Mechanistically, this phenotype can be attributed to the downregulation of the fusion protein MFN1 and the accumulation of DRP1 phosphorylated on Ser616, an activating posttranslational modification [26]. While such phosphorylation in Ras-transformed cells was anticipated, given that in Ras-driven cancers the downstream kinase Erk2 phosphorylates DRP1 at Ser616 to facilitate tumor growth [31], it was predominantly evident in the highly metastatic CA1a cells, even though AT1 and CA1h are also Ras-transformed. Thus, the contribution of MFN1 downregulation to the observed mitochondrial fragmentation warrants consideration. Phosphorylation of MFN1 by Erk1 inhibits its mitochondrial tethering and fusion ability [32], suggesting that in Ras-driven tumors, the combined phosphorylation of DRP1 and MFN1 by Erk may underlie the mitochondrial fragmentation commonly observed in metastatic cells [10, 13].

Surprisingly, levels of the pro-fusion protein OPA1 are increased in CA1a cells, where mitochondria are the most fragmented. Elevated OPA1 levels have been observed in multiple cancers and in breast cancer they are associated with worse prognosis [17] as well as with relapse upon chemotherapy [18]. Since OPA1 requires MFN1 to drive mitochondrial fusion [33], its upregulation appears ineffective in sculpting mitochondrial morphology in the context of the observed reduced MFN1 levels. Instead, increased OPA1 levels result in narrower cristae with tighter CJs, a function independent of OPA1 pro-fusion activity [15]. We predicted that these ultrastructural changes would favor mitochondrial respiration, which was indeed increased in CA1a cells despite a reduction in mtDNA levels, possibly secondary to chronic inhibition of mitochondrial fusion. Tighter cristae mediated by OPA1 upregulation favor the efficiency of the mitochondrial respiratory chain and the proliferation of cells that rely predominantly on mitochondria for ATP biosynthesis [6]. Indeed, deletion or pharmacological inhibition of OPA1 lowers mitochondrial respiration in all cell lines, except for the non-transformed 10A cells, further reinforcing the role of OPA1 as a crucial regulator of mitochondrial energy conversion in transformed cells.

While OPA1 deletion or inhibition reduces mitochondrial respiration across all transformed cell lines, it limits proliferation and migration only in the metastatic CA1h and CA1a cells. Although reduced migration or proliferation could result from cell death, we excluded this possibility within the timeframe of our analyses, suggesting an alternative mechanism whereby inhibition of this mitochondrial protein selectively reduces these key metastatic parameters. In a panel of TNBC cell lines, silencing or inhibition of OPA1 leads to upregulation of miRNAs of the 148/152 family [17]. These miRNAs inhibit tumor growth and invasiveness [34] and likely contribute to the selective effects of OPA1 deletion/inhibition on the metastatic properties of CA1a and CA1h cells.

OPA1 participates in resistance against the tyrosine kinase inhibitor gefitinib in lung adenocarcinoma [35], the Bcl-2 antagonist Venetoclax in acute myeloid leukemias [36], and in the development of residual disease following standard-of-care treatment for TNBC [18]. In all these instances, the prototypical OPA1 inhibitor MYLS22, discovered in our laboratory, has proven effective in counteracting chemoresistance. Our results extend these findings by demonstrating that OPA1 inhibition using either MYLS22 or the second-generation inhibitor Opitor-0 [21] can selectively limit the growth and migration of highly aggressive TNBC cells while sparing normal breast epithelial cells. Indeed, MYLS22 has been shown to suppress tumor growth in models of breast cancer, melanoma, and lung adenocarcinoma, with no detectable off-target toxicity in vivo [17, 19, 35], including in hematological malignancies where it displays selective anti-leukemic activity while sparing normal hematopoietic cells in vivo [37]. Notably, MYLS22-sensitive tumors consistently exhibit elevated OPA1 expression, a feature we now report in metastatic TNBC, suggesting a potential therapeutic window for targeting this pathway in vivo. Thus, our work reinforces the potential benefit of targeting OPA1 in metastatic TNBC, to limit metastatization or to eradicate residual disease following conventional therapy.

## Supplementary information

Supplementary material

Original Uncropped Western Blots

## Data Availability

Source data for all the figures are provided as a Supplementary Dataset for this paper. Reagents generated in this paper are available from the corresponding author, LS (luca.scorrano@unipd.it), upon completion of a Material Transfer Agreement.
