# Supplementary material for: Metastatic breast cancer cells are selectively dependent on the mitochondrial cristae-shaping protein OPA1
Source: Cell Death Dis. 2025 Jul 21;16(1):539. doi: 10.1038/s41419-025-07878-5 (PMC12279937; doi:10.1038/s41419-025-07878-5)
Supplement: Supplementary file 1 — Supplementary material [file 41419_2025_7878_MOESM1_ESM.pdf]

Metastatic breast cancer cells are selectively dependent on the mitochondrial cristae-shaping protein OPA1

Antigoni Diokmetzidou et al.

Supplementary Information

Supplementary Materials

Table 1. Antibodies used for Western Blotting

Antibody	Company	Cat. No.	Ab dilution	2ary Ab
DRP1	BD Biosciences	611113	1:1000	HRP goat anti-mouse IgG
FIS1	Proteintech	109561ap	1:1000	HRP goat anti-rabbit IgG
MFF	Proteintech	17090-1-AP	1:1000	HRP goat anti-rabbit IgG
MFN1	Cell Signaling Technology	14739S	1:1000	HRP goat anti-rabbit IgG
MFN2	Abnova	h409927m03	1:1000	HRP goat anti-mouse IgG
OPA1	Abcam	ab42364	1:1000	HRP goat anti-rabbit IgG
Phospho-DRP1 (Ser616)	Cell Signaling Technology	3455s	1:500	HRP goat anti-rabbit IgG
SDHA	Abcam	ab14715	1:2500	HRP goat anti-mouse IgG
TIM23	BD Biosciences	611223	1:1000	HRP goat anti-mouse IgG
TOM20	Proteintech	11802-1-AP	1:2000	HRP goat anti-rabbit IgG
TOM70	Proteintech	14528-1AP	1:1000	HRP goat anti-rabbit IgG
Vinculin	Sigma-Aldrich	V9264	1:2500	HRP goat anti-mouse IgG

Table 2. Quantitative real-time PCR primers

qPCR primers	Sequence (5’-3’)
hGAPDH-Frw	TTGGCTACAGCAACAGGGTG
hGAPDH-Rev	GGGGAGATTCAGTGTGGTGG
hND1-Frw	ATGGCCAACCTCCTACTCCT
hND1-Rev	TAGATGTGGCGGGTTTTAGG

Supplementary Figures

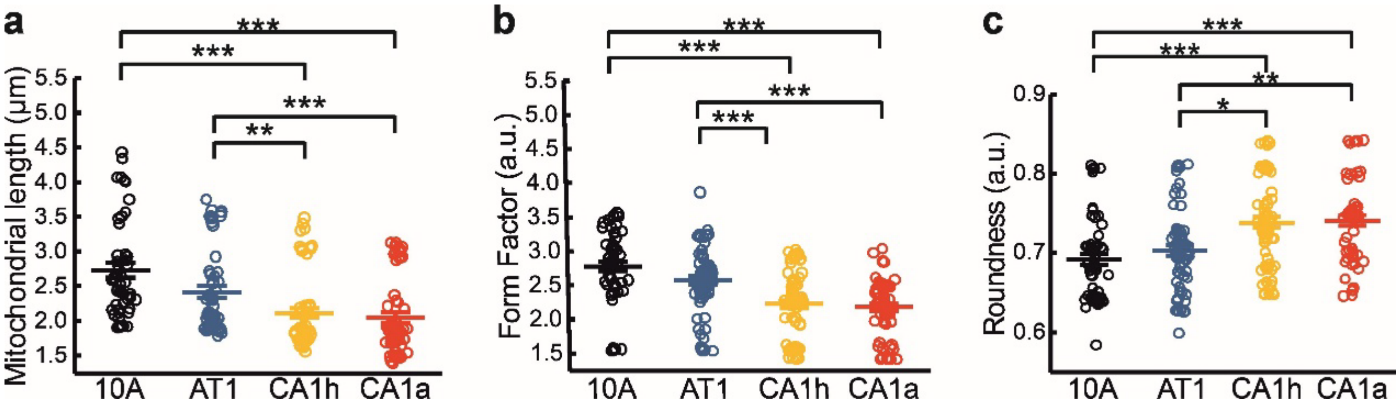

Supplementary Figure S1. Mitochondrial are fragmented in the metastatic cells.
a-c Average±SEM of mitochondrial length (**a**), form factor (**b**) and roundness (perimeter2/4π*area; **c**) in the indicated cells from n=7 biological replicates of experiments as in Figure 1a. Each dot refers to the mean of ≥40 imaging fields per well. *p <0.05, **p <0.01 ***p< 0.001 in a Kruskal-Wallis ANOVA with Dunn's post-hoc test between the indicated conditions.

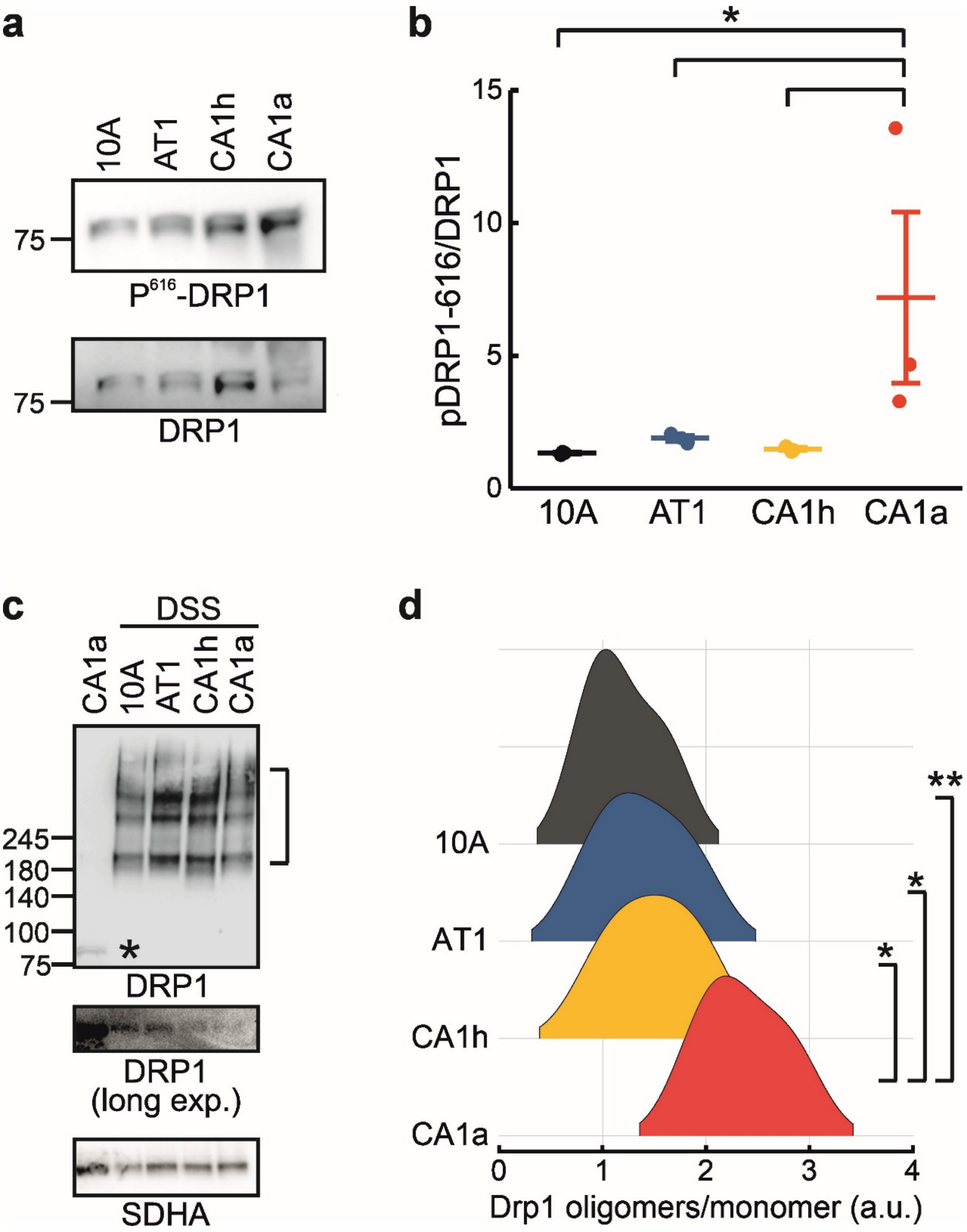

Supplementary Figure S2. DRP1 is activated in metastatic breast cancer cells.
a Cells were lysed and equal amounts of proteins (30 µg) were separated by SDS-PAGE and immunoblotted using the indicated antibodies.
b Quantification by densitometry of the Phospho-Ser616 to total DRP1 ratio in n=3 biological replicates of experiments as in a. *p ≤0.05 in a One-way ANOVA with Fisher's post-hoc test between the indicated conditions.
c The indicated cells were treated where indicated with DSS (1mM, 1h) lysed. Equal amounts of proteins (30 µg) were separated by SDS-PAGE and immunoblotted using the indicated antibodies. Asterisk: monomeric DRP1; bracket: DRP1 oligomers. Long exp.: longer exposure of monomer immunoblot region.
d Ridgeline chart of densitometric analysis of DRP1 oligomers/monomer ratio in n=3 biological replicates of experiments as in c. *p <0.05, **p <0.01 in One-way ANOVA with Fisher's post-hoc test between the indicated conditions.

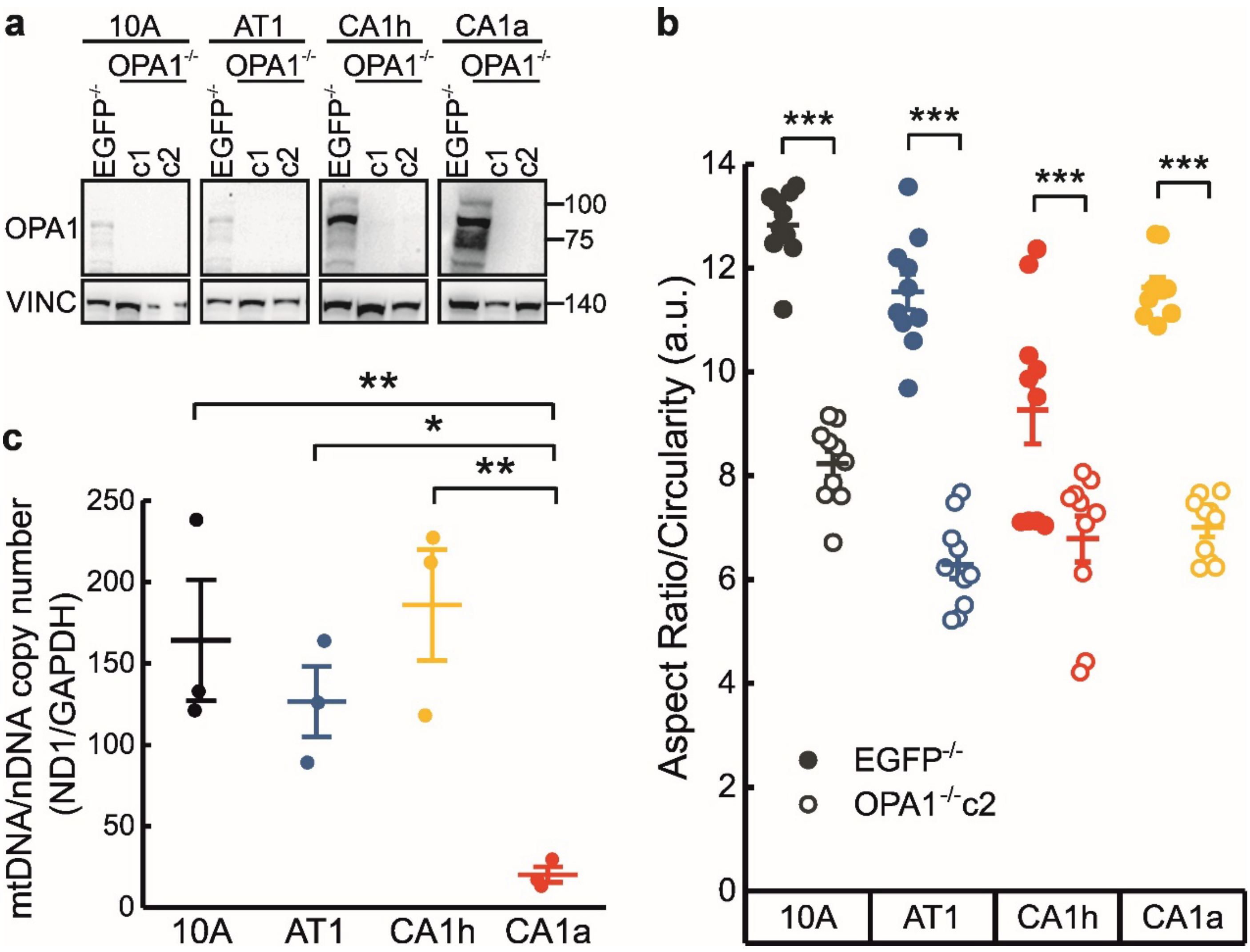

Supplementary Figure S3. Deletion of OPA1 in the TNBC cells fragments mitochondria.
a Cells were lysed and equal amounts of proteins (30 µg) were separated by SDS-PAGE and immunoblotted using the indicated antibodies (representative from n=3 biological replicates).
b Mean±SEM of mitochondrial aspect ratio (length/width) normalized by circularity in cells of the indicated genotype stained with Mitotracker Deep Red as in Figure 3a. Each dot refers to the mean of ≥40 imaging fields/well from n=6 biological replicates. *p≤0,05, **p< 0,01, ***p< 0,001 in a One-way ANOVA with Tukey's post-hoc test between the indicated conditions.
c mtDNA/nuclear DNA copy number ratio measured by qPCR. Data represent the mean ± SEM of 3 biological replicates; *p ≤ 0.05, **p <0.01 in a One-way ANOVA with Fisher's post-hoc test between the indicated conditions.
