# Supplementary figures and images for: Metastatic breast cancer cells are selectively dependent on the mitochondrial cristae-shaping protein OPA1
Source: Cell Death Dis. 2025 Jul 21;16(1):539. doi: 10.1038/s41419-025-07878-5 (PMC12279937; doi:10.1038/s41419-025-07878-5)

**Original Western Blots**

**Figure 1**


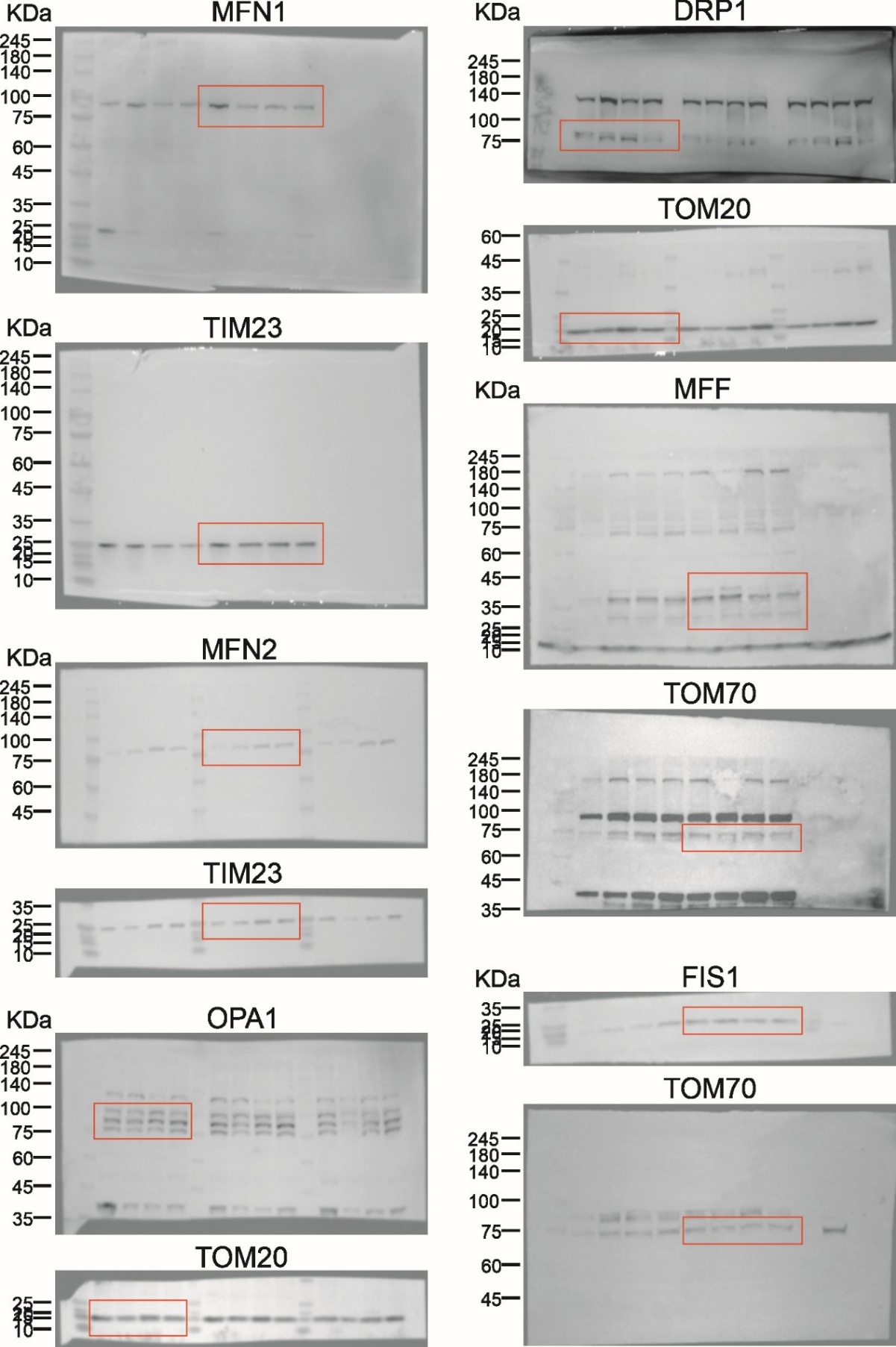


**Figure 2**

**
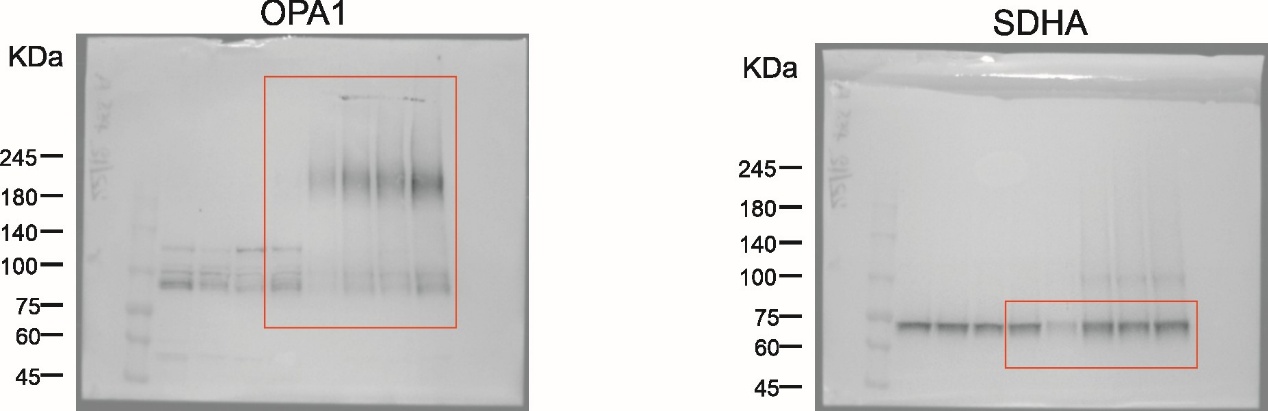
**

**Figure S2**

**
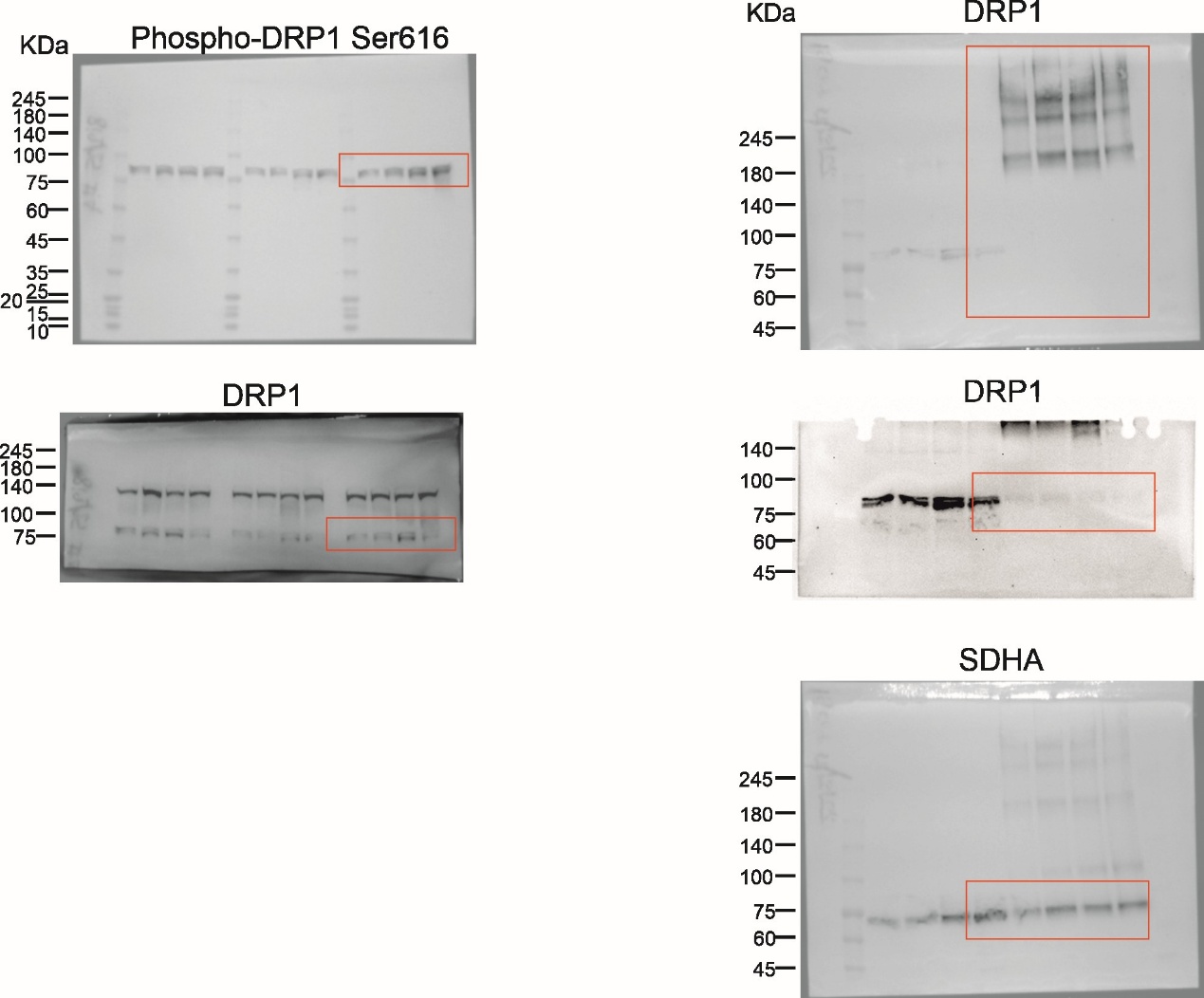
**

**Figure S3**

**
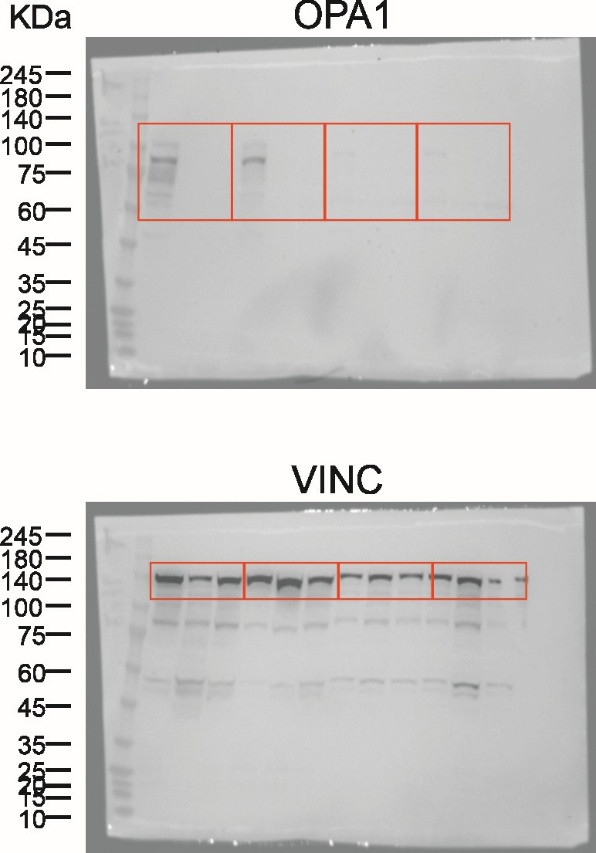
**

Supplement: Supplementary file 2 — Original Uncropped Western Blots [file 41419_2025_7878_MOESM2_ESM.docx]
